# Characteristics Associated with Mental and Physical Health Among US Adults with Long COVID

**DOI:** 10.3390/healthcare14152430

**Published:** 2026-08-06

**Authors:** David R. Axon, Regan F. Szott

**Affiliations:** James L. Winkle College of Pharmacy, University of Cincinnati, 3255 Eden Ave., Cincinnati, OH 45267, USA; szottrf@mail.uc.edu

**Keywords:** long COVID, mental health, health status, medical expenditure panel survey

## Abstract

**Background/Objectives:** Long COVID (LC) has affected 7.2% of the population of the United States (US). Mental and physical health have been increasing in prevalence over the last few years. This study aimed to investigate the association between various characteristics and mental and physical health status among US adults with LC. **Methods:** The study was cross-sectional in design and used data from the 2023 Medical Expenditure Panel Survey (MEPS). We assessed predisposing, enabling, and need variables in US adults with MEPS-defined LC using multivariable logistic regression analysis. The data was weighted to produce nationally representative estimates. **Results:** It was determined that individuals with a low income level, a high degree of pain, and poor physical health were each associated with higher odds of poor mental health in US adults with LC. An age of 50–70+ was associated with lower odds of poor physical health in US adults with LC. Educational achievement up to and including high school, having a functional limitation, exercise participation, any pain, multiple comorbid conditions, and poor mental health were each associated with higher odds of poor physical health in US adults with LC. **Conclusions:** Several variables were associated with poor mental and physical health status among US adults with LC. Further research should be conducted to explore these variables in more detail and investigate possible interventions for healthcare providers.

## 1. Introduction

Long COVID (LC) is defined as a chronic condition that is present for at least three months after an initial SARS-CoV-2 infection per the United States (US) Centers for Disease Control and Prevention [1]. LC has recently been found to affect 7.2% of the US population [2]. Symptoms can range from fatigue, dyspnea, cough, renal disease, and, in severe cases, neurological symptoms [3,4]. LC has been associated with increased work impairment and unemployment, leading to nearly 1 million US adults being out of work due to LC [5,6] and causing the US to lose an estimated $170 billion in earnings in 2023 [7].

Aside from LC, poor mental health is increasingly prevalent across the US, with nearly 1 in 5 individuals in the US population having some sort of mental illness in 2022 [8]. One report highlighted a 38.8% increase in mental health services after the COVID-19 pandemic [9]. The literature has shown that individuals who had COVID-19 are at a higher risk of developing mental health problems [10]. This warrants further review of the factors associated with poor mental health in US adults with LC.

In addition, poor physical health is also common in the US population. Approximately 13.6% of the US population categorized themselves as being in poor health per a 2021 National Health Interview Survey [11]. A previous study has shown that adults with LC are likely to experience physical health challenges post exposure [12]. However, there is a lack of studies directly exploring the factors that are associated with poor physical health among US adults with LC.

There has been an increase in the published literature exploring the impact of LC on mental and physical health. For instance, a cross-sectional database study in the US found that having LC was associated with higher risk of anxiety and depressive symptoms [13]. Outside the US, a study using data from a cohort of patients in England found that depressive and anxiety symptoms increased after the onset of LC [14]. A systematic review and meta-analysis found several psychological factors were associated with LC, including depression and anxiety [15]. Another scoping review suggested that individuals with LC and existing mental health conditions may have an increased risk of worsening mental health [16]. A further systematic review of the impact of LC on the health of adults with pre-existing cardiovascular disease and hypertension found a variety of studies reporting prolonged LC symptoms, physiological health outcomes, lifestyle behaviors, psychosocial outcomes, cardiovascular disease complications, hospital readmissions, and death [17]. Another study demonstrated that adults with LC had significantly higher pain, more comorbidities, more fatigue, lower health-related quality of life, and lower sleep quality compared to individuals without LC, and that individuals with LC experienced enduring physical and health-related challenges that negatively impacted their overall health and well-being [12]. One further study of patients in California found that adults with LC had greater anxiety, depression, physical function limitations, fatigue, social limitations, activity limitations, and pain interference than adults without LC [18].

However, since the COVID-19 outbreak in 2020, there has been limited investigation of the relationships between long-term symptoms following an initial SARS-CoV-2 infection, i.e., LC, and the mental and physical health status of the US adult population. As LC is increasingly recognized and diagnosed, researchers must understand the relationship between various factors and mental and physical health to help treat patients with LC and to help ensure that the highest quality of care and resources are provided. The objectives of this study were to identify the variables associated with: (1) poor versus good mental health and (2) poor versus good physical health, among US adults with LC. Identification of the variables associated with poor mental health and poor physical health may help improve our understanding of this new condition, suggest areas for further investigation, and may indicate areas where interventions could be made to improve health outcomes.

## 2. Materials and Methods

### 2.1. Study Design and Data Source

This was a cross-sectional study conducted using the 2023 Medical Expenditure Panel Survey (MEPS) dataset. MEPS is a set of large-scale surveys that collect demographic and healthcare data on adults in the US. Three components are currently used for data reporting in MEPS: the Household Component (MEPS-HC), the Medical Provider Component (MEPS-MPC), and the Insurance Component (MEPS-IC). MEPS-HC collects data from individual household members, while MEPS-MPC and MEPS-IC collect data from medical providers and insurance providers, respectively. Data from MEPS-MPC and MEPS-IC are used to supplement the data collected in MEPS-HC. By oversampling disabled and minority groups, MEPS can supply estimates of the nationally representative noninstitutionalized US population. MEPS is carried out by the Agency for Healthcare Research and Quality and is annually authorized by an Institutional Review Board. This study utilized the 2023 full-year consolidated data file, which provides data on 1374 variables for the 2023 calendar year [19,20,21].

### 2.2. Eligibility Criteria and Long COVID

Eligibility criteria for this study were US adults over the age of 18 who had MEPS-defined LC and survived the full calendar year. MEPS-defined LC was identified from an item in the priority conditions enumeration section of the MEPS. In this item, participants are asked if they have ever been diagnosed with COVID-19. If an individual responds positively to this item, then the individual is asked if they had symptoms lasting three months or longer that they did not have prior to having COVID-19 (variable name = LCEVER53). Individuals who said yes to this item were defined as having LC by MEPS [19,20].

### 2.3. Dependent Variable 1: Mental Health

The first of two dependent variables assessed in this study was mental health status. This dichotomous variable (poor versus good mental health) was determined from an MEPS item that asked participants to rate their mental health (variable name = MNHLTH31/42/53).

### 2.4. Dependent Variable 2: Physical Health

The second dependent variable assessed in this study was physical health status. This dichotomous variable (poor versus good mental health) was determined from an MEPS item that asked participants to rate their health (variable name = RTHLTH31/42/53).

### 2.5. Independent Variables

The independent variables in this study were conceptualized using the Andersen Behavioral Model (ABM) [16]. The ABM was developed in the 1960s and has been updated and adapted for various health service research needs ever since. In particular, the ABM may be used to organize variables in a model assessing (health) outcomes. For the purposes of this study, three aspects of the ABM were used: predisposing variables, enabling variables, and need variables [22].

The predisposing variables in this study included age (variable name = AGE23X), sex (variable name = sex), race (variable name = RACEV1X), and ethnicity (variable name = HISPANX) [20,21].

The enabling variables in this study included marital status (variable name = MARRY23X), education achieved (variable name = EDUCYR), employment status (variable name = EMPLOY), income level (variable name = POVCAT23), and insurance coverage (variable name = INSCOV23) [20,21].

The need variables in this study included functional limitation (variable name = WLKLIM31), exercise (variable name = PHYEXE53), smoker status (variable name = OFTSMK53), pain interference (variable name = ADPAIN42), and number of comorbid conditions (from the following list: hypertension (variable name = BPMLDX), coronary heart disease (variable name =CHDDX), angina (variable name = ANGIDX), myocardial infarction (variable name = MIDX), other heart disease (variable name = OHRTDX), stroke (variable name = STRKDX), emphysema (variable name = EMPHDX), chronic bronchitis (variable name = CHBRON31), high cholesterol (variable name = CHOLDX), cancer (variable name = CANCERDX), diabetes (variable name = DIABDX_M18), joint pain (variable name = JTPAIN31_M18), arthritis (variable name = ARTHDX), and asthma (variable name = ASTHDX)). In the model where mental health was the dependent variable, physical health (variable name = RTHLTH31/42/53) was also included as a need variable. In the model where physical health was the dependent variable, mental health (variable name = MNHLTH31/42/53) was also included as a need variable [20,21].

### 2.6. Data Analysis

SAS (v.9.4, SAS Institute Inc., Cary, NC, USA) software and the associated survey procedures (e.g., SURVEYFREQ, SURVEYLOGISTIC) were used for data analysis. Differences between groups for each characteristic were compared with a chi-square test. Two multivariable logistic regression models were constructed to assess the association between each independent variable and (1) poor versus good mental health status and (2) poor versus good physical health status. Multicollinearity was assessed by examining the variance inflation factor and found not to be significant. A significance level of α < 0.05 was selected a priori. Cluster (variable name = VARPSU) and stratum (variable name = VARSTR) variables were used to maintain the integrity of the complex survey data. A weighting variable (variable name = SAQWT23F) was used to get nationally weighted estimates. The predictive ability of each model was assessed by examining the c-statistics (with a higher value indicating stronger predictive ability). The University of Cincinnati Institutional Review Board approved this study (protocol number: 2026-0518, approval date: 11 May 2026).

## 3. Results

### 3.1. Study Sample and Weighted Population

**Figure 1:**
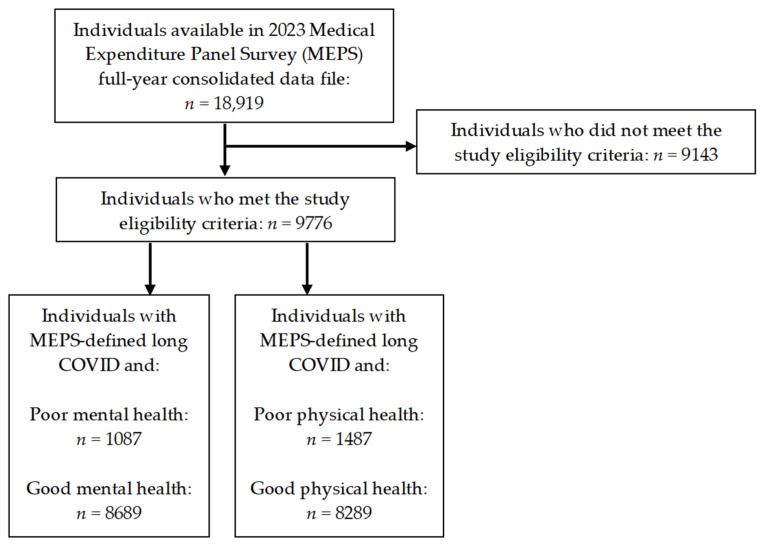
Study eligibility flowchart.

A total of 9776 individuals (weighted n = 258,426,862) with LC met the inclusion criteria of the study and were included in the analysis. Of these, 1087 reported poor mental health (weighted n = 27,717,031), and 8689 (weighted n = 230,709,831) reported good mental health. Meanwhile, 1487 reported poor physical health (weighted n = 33,733,701), and 8289 (weighted n = 224,693,161) reported good physical health, see Figure 1.

### 3.2. Baseline Characteristics

The baseline characteristics of adults with LC in the US stratified by poor versus good mental health and poor versus good physical health are reported in Table 1. These results showed there were significant differences between groups in both the mental health and physical health samples according to marital status, employment status, income level, insurance coverage, functional limitation, exercise participation, pain interference, comorbid conditions, and physical health (in the mental health sample) or mental health (in the physical health sample). In addition, there was a significant difference between groups for education in the physical health sample.

### 3.3. Variables Associated with Poor Mental Health

Table 2 shows the variables associated with poor mental health in US adults with LC. Individuals with poor/low versus middle/high income levels, extreme/quite a bit versus no pain interference, and poor versus good physical health were each associated with higher odds of poor mental health in US adults with LC.

### 3.4. Variables Associated with Poor Physical Health

Table 2 also shows the variables associated with poor physical health in US adults with LC. Age 70+, 60–69, and 50–59 versus 18–29 years were each associated with lower odds of poor physical health in US adults with LC. Educational achievement up to and including high school education versus more than high school education; having a functional limitation yes versus no; no regular exercise participation versus regular exercise participation; extreme/quite a bit, moderate, or little versus no pain interference; 4+ or 2–3 versus 0–1 comorbid conditions; and poor versus good mental health were each associated with higher odds of poor physical health in US adults with LC.

## 4. Discussion

### 4.1. Summary of Key Findings

The key findings of this study were that several need variables were associated with poor mental or physical health in US adults with LC, including higher levels of pain interference in both models, having poor physical health in the mental health model, and having a functional limitation, not participating in exercise, having pain interference, having multiple comorbid conditions, and poor mental health in the physical health model. In addition, one enabling variable, income level, was associated with poor mental health, while education achieved was associated with poor physical health. Among the predisposing variables, only older age was associated with poor physical health. The other variables investigated in this study were not associated with poor mental or physical health in US adults with LC. These findings are further discussed in detail below, beginning with the need variables, as these offer the greatest opportunity to address or make interventions.

### 4.2. Need Variables

Extreme/quite a bit vs. no pain interference was associated with higher odds of reporting poor mental health and poor physical health among US adults with LC. Moderate and low levels of pain interference were also associated with poor physical health, but were not associated with poor mental health in this study. These are reasonable findings supported by the existing literature. Previous work has established that there is a bidirectional relationship between pain and mental health [23]. Specifically, among people with LC, a previous study found that those with pre-existing mental health disorders had more severe LC symptoms [24]. The same study also showed that mental health rates were increasing post COVID-19 [24]. Likewise, with respect to physical health, it is reasonable that those who are in pain also have poorer health and are perhaps less likely to participate in activities that improve physical health [24]. A previous study found that three out of ten people with LC report being in pain, which leads to negative health outcomes [25]. On the other hand, adults with LC may have been experiencing pain before contracting LC. Pain itself is associated with poor physical health outcomes, as seen in a study that found that those suffering from pain reported significant impairment in completing daily activities and physical functioning [26]. Conversely, it may be the case that having poor health leads to pain. It is logical to observe an association between pain and mental or physical health status, but it is not possible from this study to determine the precipitating factor; i.e., did pain occur before poor mental or poor physical health, or was poor mental and physical health established before pain? Further research exploring pain interference and mental and physical health status with a causal study design, specifically among patients with LC, may be warranted.

Poor physical health was associated with higher odds of reporting poor mental health in US adults with LC. Similarly, poor mental health was associated with higher odds of reporting poor physical health in this analysis. These are unsurprising findings given the established relationship between these variables in the general population [27]. Without regard to LC, it is known that there is a prevalent association between poor mental health and poor physical health [28]. This could be due to a lack of motivation to care for oneself, as seen in depression and other diseases, among other reasons. Interestingly, there is an association between patients with severe mental illness and undiagnosed physical conditions. A study has previously discussed that this may be due to a lack of physical care access and not seeking care due to symptoms of mental illness [29]. One of the most common symptoms of LC is fatigue, which is related to chronic fatigue syndrome [30,31]. Chronic fatigue syndrome is indicated by debilitating tiredness that is not alleviated by rest and is often worsened by physical exertion. It is known to lead to health decline through organ dysfunction and immune dysregulation [32]. Having chronic fatigue due to LC can lead to mental health decline as well as physical health decline [33]. The previous literature has also shown that higher-severity LC has been linked to those with long-term mental health issues [16,34]. In addition, one of the many symptoms of LC is post-exertional malaise. Physical therapists supporting adults with LC should monitor fatigue and exertional malaise as they work with patients to improve their physical health [35]. A recent systematic literature review indicated that 24 trials were ongoing related to drug or non-drug interventions for LC and concluded that there was moderate-certainty evidence that cognitive behavioral therapy and physical and mental health rehabilitation improve LC symptoms [36].

This study also showed an association between the number of chronic conditions and physical health status among US adults with LC. Individuals with more conditions were associated with greater odds of reporting poor physical health. This is reasonable, as multiple conditions can have additive effects, impacting overall health [37]. These additional conditions, on top of LC, would logically be associated with poorer physical health. Likewise, it may be the case that having poorer physical health contributes to the number of chronic conditions. The existing literature has shown that those who had pre-existing respiratory conditions were more likely to suffer from long-lasting symptoms of COVID-19 [38]. Of note, MEPS includes respiratory conditions associated with LC, such as asthma, emphysema, and chronic bronchitis, in the list of chronic conditions. A cross-sectional study showed that more chronic conditions leads to poorer quality of life, including poor physical health outcomes. Although this study’s sample was adults over the age of 50 across the globe and is therefore less representative of the current study’s population, the correlation is apparent [39].

The association between having a functional limitation and reporting poorer physical health observed in the current study is also a reasonable finding. Previous studies have shown that LC can lead to a functional limitation [40,41]. Functional limitations arising from LC may lead to difficulty completing daily tasks, which, in turn, result in poorer physical health. Conversely, patients may have had a functional limitation before LC, which could stem from multiple reasons, such as previous stroke, osteoarthritis, or other events [42]. These conditions alone already leave a patient in poor physical health, and with the addition of LC, functional status may decline, also decreasing physical health.

Furthermore, this study found that those who reported not exercising regularly were associated with increased odds of reporting poor physical health. This is feasible, as many studies show the correlation between exercising regularly and better physical health [43]. It is also reasonable to assume that those with severe LC symptoms may be less likely to partake in exercise due to their LC symptoms overall, leading to poorer physical health.

Smoking status was not associated with poor mental or physical health. This is interesting given that the previous literature has shown some association with smokers contracting worse symptoms from LC and, therefore, being in poorer health [44].

### 4.3. Enabling Variables

One enabling variable (income level) was associated with mental health, while a different enabling variable (education achieved) was associated with physical health. A low-income status was associated with higher odds of reporting poor mental health in the current study. This is a logical finding as financial struggles are a leading cause of mental health stress in US adults [45]. Studies have also shown that those with low income may struggle to gain access to healthcare services, which increases their risk of experiencing long-term complications from an initial COVID infection [46]. Based on the findings from the current study, it is possible that unplanned healthcare costs when experiencing LC symptoms may contribute to poorer mental health. Conversely, it is possible that pre-existing poor mental health leads to lower income and thus higher financial burden. Alternatively, those with poor mental health and low income may be unable to seek the therapy they need, and the finding in the current study simply reflects their baseline status before LC.

Those who had a high school education or lower were associated with higher odds of reporting poor physical health in the current study. A previous study found that those who had higher education levels lived longer than their less-educated peers, possibly due to greater reasoning skills that could be utilized in healthcare decisions [47]. Another study found an association between educational achievement and the intensity of COVID-19 symptoms, where lower educational achievement was linked to more intense COVID-19 symptoms [10]. Greater emphasis on interventions for mental health may be warranted for lower-income individuals, while more interventions to improve physical health may be useful for those with lower educational achievement.

### 4.4. Predisposing Variables

Among the predisposing factors, only the age groups older than 50 (i.e., 50–59, 60–69, and 70+) versus 18–29 years were associated with lower odds of reporting poor physical health in this study. Previous studies have shown different results, where older adults across the world with LC are in poorer health [48,49]. One possible explanation for the finding in the current study is that there may be differences in how younger and older adults perceive the burden of LC symptoms. Likewise, as people age, they may expect to have poorer health, but if it is better than they expect, they may be biased in their responses. Another possible explanation is that some older adults who had LC have died, and only those with milder symptoms were alive to respond to the survey (i.e., survivor bias). Alternatively, those with more severe LC may have been institutionalized because of their condition (e.g., hospitalized, living in a managed care facility), and therefore not included in the MEPS, which only captures data for noninstitutionalized individuals. Further research is needed to investigate the role of aging in LC and to understand the reasons why there may be differences in health status by age.

These findings indicate a need to investigate mental and physical health specifically among younger adults, which may be overlooked. One US study reported that younger adults (aged 18–44) and middle-aged adults (aged 45–64) had poorer neurological symptoms than older adults (aged ≥65), suggesting the need for additional support for younger adults with LC [50]. Another study showed LC was associated with increased odds of elevated psychological distress among young adults in the US [51]. Outside the US, research has shown several poor health aspects among younger adults following COVID-19 infection. For example, one study from Slovakia of young adults aged 18–30 showed there were several long-term consequences of COVID-19 infection among younger female adults, including difficulty with cognitive tasks, memory deterioration, and concentration problems, as well as physiological symptoms in both males and females, such as decreased physical fitness [52]. Another Slovak study using a similar study sample found that the COVID-19 pandemic had a negative effect on bone mass density and bone mineral content, which may indicate poor physical health among these young adults and suggests LC is part of a multisystem disorder [53].

There was no significant association between the other predisposing factors and poor mental or physical health in this study. Of note, the older age categories were not associated with poor mental health in this analysis. This is interesting, as previous research has found that among the general population, as people age, they are more likely to experience life changes that negatively impact their mental health [54]. Sex, race, and ethnicity were not associated with reporting poorer mental/physical health in LC.

### 4.5. Limitations and Future Work

One study limitation is the cross-sectional nature of the study design, which can only demonstrate a statistical association and not causality. Additionally, LC was a new disease when the MEPS data were collected in 2023, and not all respondents may have had a clear understanding of it. Other limitations include the use of self-reported data, which may be subject to recall and other biases, as well as different definitions of LC, mental health status, and physical health status. In this study, LC was defined using a single (binary) item. Given the complex nature of LC, future research may benefit from examining different profiles or clusters of symptoms to determine if there are variations in the association with mental and physical health. Although the MEPS dataset contains many variables, it lacks specific data on various additional aspects of COVID. For instance, the dataset does not capture the severity of the initial acute COVID-19 infection, time since infection, number of re-infections, any health service utilization because of COVID-19 infection (e.g., hospitalization or intensive care unit admission), the COVID-19 variant contracted, or details on the number and timing of COVID-19 vaccinations. Each of these variables may be confounders for LC and may be associated with mental and physical health, which should be accounted for in future research. Given that there may be different manifestations in LC between various characteristics, e.g., age or sex, future work may consider interaction terms or stratification of these characteristics. Furthermore, although multicollinearity was not detected in the model, some included variables (e.g., functional limitations and pain interferences) are conceptually similar to physical health, which may have led to over-adjustment of the model.

## 5. Conclusions

This database study found that several need variables were associated with mental health and physical health among US adults with LC, while only one enabling variable was associated with mental health or physical health and only older age was associated with mental health. Further research is needed to investigate these factors in greater detail and to understand the mechanisms that underly these observations. Further research could also explore symptom-specific pathways, longitudinal trajectories, interactions between variables and mental/physical health, and stratification to detect any differences in mental/physical health based on specific demographic characteristics. It is also important to ensure appropriate integrated and mental health support is provided for patients with LC [24].

## Figures and Tables

**Table 1:** Characteristics of adults with long COVID from the United States stratified by mental and physical health status.

Variable	Poor Mental Health % (95% CI)	Good Mental Health % (95% CI)	*p*	Poor Physical Health % (95% CI)	Good Physical Health % (95% CI)	*p*
**Predisposing variables**						
Age			0.4288			0.8765
70+	10.8 (5.4–16.3)	12.3 (9.8–14.8)		10.5 (6.3–14.6)	12.5 (9.8–15.2)	
60–69	19.6 (12.0–27.2)	17.7 (14.8–20.7)		18.2 (11.5–24.9)	18.0 (14.9–21.1)	
50–59	20.0 (14.4–25.6)	22.3 (17.9–26.6)		26.0 (18.8–33.1)	20.8 (16.8–24.8)	
40–49	11.9 (6.8–17.1)	17.2 (14.5–19.9)		16.2 (10.3–22.1)	16.3 (13.5–19.2)	
30–39	25.1 (17.1–33.1)	18.3 (15.0–21.6)		17.7 (10.1–25.3)	19.9 (16.1–23.6)	
18–29	12.6 (5.4–19.8)	12.2 (9.0–15.5)		11.4 (3.8–19.0)	12.5(9.3–15.7)	
Sex			0.0904			0.4386
Male	35.7 (27.4–44.1)	44.0 (39.8–48.2)		39.9 (32.2–47.6)	43.3 (39.1–47.6)	
Female	64.3 (55.9–72.6)	56.0 (51.8–60.2)		60.1 (52.4–67.8)	56.7 (52.4–60.9)	
Race			0.6676			0.5429
White	83.0 (75.6–90.5)	82.7 (79.3–86.0)		83.8 (76.8–90.9)	82.4 (79.2–85.7)	
Black	8.6 (4.7–12.5)	7.4 (5.3–9.4)		9.5 (4.2–14.8)	7.0 (5.0–9.1)	
Asian	2.6 (0.0–7.3)	5.4 (3.2–7.6)		2.6 (0.0–5.3)	5.6 (3.1–8.0)	
Other/multiple	5.8 (0.8–10.7)	4.6 (2.8–6.3)		4.1 (0.0–9.1)	5.0 (2.8–7.1)	
Ethnicity			0.0663			0.2497
Hispanic	13.3 (8.1–18.5)	18.9 (15.2–22.6)		14.6 (8.6–20.6)	18.9 (14.9–22.9)	
Non-Hispanic	86.7 (81.5–91.9)	81.1 (77.4–84.8)		85.4 (79.4–91.4)	81.1 (77.1–85.1)	
**Enabling variables**						
Marital status			0.0002			0.0089
Married	38.0 (28.7–47.3)	58.0 (53.7–62.3)		44.3 (35.6–53.0)	57.5 (53.1–61.8)	
Not married	62.0 (52.7–71.3)	42.0 (37.7–46.3)		55.7 (47.0–64.4)	42.5 (38.2–46.9)	
Education achieved			0.1080			0.0202
Up to and including high school	52.2 (48.0–56.5)	39.1 (37.1–41.1)		49.9 (41.5–58.4)	38.6 (34.0–43.1)	
More than high school	47.8 (43.5–52.0)	60.9 (58.9–62.9)		50.1 (41.6–58.5)	61.4 (56.9–66.0)	
Employment status			0.0224			0.0102
Employed	55.5 (44.6–66.5)	67.2 (62.9–71.5)		55.6 (46.0–65.3)	67.8 (63.3–72.3)	
Unemployed	44.5 (33.5–55.4)	32.8 (28.5–37.1)		44.4 (34.7–54.0)	32.2 (27.7–36.7)	
Income level			<0.0001			0.0134
Poor/low	49.6 (39.6–59.5)	23.1 (19.6–26.6)		36.8 (28.4–45.1)	25.0 (20.9–29.2)	
Middle/high	50.4 (40.5–60.4)	76.9 (73.4–80.4)		63.2 (54.9–71.6)	75.0 (70.8–79.1)	
Insurance coverage			<0.0001			0.0065
Private	54.5 (49.6–59.4)	68.2 (66.2–70.2)		56.4 (47.5–65.3)	69.9 (65.6–74.1)	
Public	41.5 (36.9–46.2)	25.0 (23.4–26.6)		39.0 (30.1–47.8)	23.9 (20.0–27.9)	
None	4.0 (2.5–5.5)	6.8 (5.7–7.9)		4.6 (0.6–8.6)	6.2 (4.0–8.4)	
**Need variables**						
Functional limitation			<0.0001			<0.0001
Yes	36.4 (28.5–44.4)	18.6 (15.3–21.8)		44.3 (36.4–52.2)	15.5 (12.3–18.6)	
No	63.6 (55.6–71.5)	81.4 (78.2–84.7)		55.7 (47.8–63.6)	84.5 (81.4–87.7)	
Exercise participation			0.0054			<0.0001
No	61.2 (53.4–69.0)	48.9 (44.7–53.0)		68.5 (61.9–75.2)	46.3 (41.9–50.7)	
Yes	38.8 (31.0–46.6)	51.1 (47.0–55.3)		31.5 (24.8–38.1)	53.7 (49.3–58.1)	
Smoker			0.1380			0.4477
Yes	15.1 (8.9–21.3)	10.5 (7.4–13.6)		13.0 (7.5–18.6)	10.8 (7.6–14.0)	
No	84.9 (78.7–91.1)	89.5 (86.4–92.6)		87.0 (81.4–92.5)	89.2 (86.0–92.4)	
Pain interference			<0.0001			<0.0001
Extreme/quite a bit	34.9 (27.3–42.6)	9.5 (6.8–12.2)		34.9 (27.3–42.5)	8.0 (5.6–10.5)	
Moderate	13.8 (8.3–19.2)	11.8 (9.1–14.5)		14.4 (9.4–19.4)	11.5 (8.8–14.2)	
Little	27.4 (18.9–35.8)	28.4 (24.1–32.6)		33.0 (24.6–41.4)	26.9 (22.9–30.9)	
None	23.9 (15.2–32.6)	50.3 (45.6–55.0)		17.7 (11.0–24.3)	53.5 (48.7–58.4)	
Comorbid conditions			0.0001			<0.0001
4+	37.6 (29.1–46.2)	23.0 (19.7–26.3)		41.3 (32.8–49.7)	21.2 (18.3–24.1)	
2–3	37.1 (29.2–44.9)	33.6 (30.0–37.3)		41.1 (32.7–49.4)	32.4 (28.6–36.2)	
0–1	25.3 (17.7–32.9)	43.4 (39.3–47.5)		17.7 (11.5–23.8)	46.4 (42.3–50.5)	
Physical health			<0.0001			N/A
Poor	57.2 (46.9–67.6)	13.9 (10.5–17.3)		N/A	N/A	
Good	42.8 (32.4–53.1)	86.1 (82.7–89.5)		N/A	N/A	
Mental health			N/A			<0.0001
Poor	N/A	N/A		45.3 (36.0–54.5)	9.1 (6.5–11.7)	
Good	N/A	N/A		54.7 (45.5–64.0)	90.9 (88.3–93.5)	

Differences between groups were determined with chi-square tests. CI = confidence interval.

**Table 2:** Variables associated with poor versus good mental health and poor versus good physical health among adults with long COVID from the United States.

Variable	Poor Versus Good Mental Health Odds Ratio (95% CI)	Poor Versus Good Physical Health Odds Ratio (95% CI)
**Predisposing variables**		
Age 70+ vs. 18–29 years	0.3 (0.1–1.2)	0.1(0.1–0.3)
Age 60–69 vs. 18–29 years	0.5 (0.1–1.6)	0.2 (0.1–0.5)
Age 50–59 vs. 18–29 years	0.3 (0.1–1.0)	0.4 (0.1–0.9)
Age 40–49 vs. 18–29 years	0.5 (0.2–1.2)	0.7 (0.3–1.8)
Age 30–39 vs. 18–29 years	1.2 (0.5–3.2)	0.5 (0.2–1.6)
Male vs. female sex	0.6 (0.4–1.1)	0.9 (0.6–1.4)
White vs. other/multiple race	1.0 (0.4–2.8)	1.5 (0.3–8.2)
Black vs. other/multiple race	0.8 (0.3–2.5)	1.8 (0.3–11.4)
Asian vs. other/multiple race	0.6 (0.1–5.9)	1.0 (0.1–14.2)
Hispanic vs. non-Hispanic	0.7 (0.4–1.3)	0.8 (0.4–1.6)
**Enabling variables**		
Married vs. not married	0.7 (0.4–1.2)	1.0 (0.6–1.6)
Up to and including vs. more than high school education achieved	1.0 (0.6–1.5)	1.6 (1.0–2.6)
Employed vs. unemployed	1.1 (0.6–2.1)	1.0 (0.6–1.7)
Poor/low vs. middle/high income level	3.1 (1.7–5.7)	0.6 (0.3–1.1)
Private vs. no insurance coverage	1.0 (0.3–3.1)	0.6 (0.1–2.2)
Public vs. no insurance coverage	0.8 (0.2–2.7)	1.0 (0.2–3.9)
**Need variables**		
Functional limitation yes vs. no	0.7 (0.4–1.2)	1.9 (1.1–3.1)
Exercise participation no vs. yes	0.8 (0.6–1.2)	2.3 (1.5–3.6)
Smoker yes vs. no	1.0 (0.5–1.8)	0.7 (0.4–1.4)
Extreme/quite a bit vs. no pain interference	4.5 (2.0–10.1)	5.1 (2.3–11.4)
Moderate vs. no pain interference	1.8 (0.9–3.7)	2.9 (1.3–6.7)
Little vs. no pain interference	1.6(0.9–2.9)	2.9 (1.5–5.6)
4+ vs. 0–1 comorbid conditions	2.2 (1.0–5.0)	4.6 (2.4–8.7)
2–3 vs. 0–1 comorbid conditions	1.5 (0.8–2.9)	2.8 (1.5–5.3)
Poor vs. good physical health	5.4 (3.0–9.7)	N/A
Poor vs. good mental health	N/A	5.3 (2.9–9.6)

Wald statistic < 0.0001. Mental health model c-statistic = 0.82. Physical health model c-statistic = 0.84. CI = confidence interval.

## Data Availability

The raw data supporting the conclusions of this article will be made available by the authors on request.

## Abbreviations

The following abbreviations are used in this manuscript:

ABM	Anderson Behavioral Model
LC	Long COVID
MEPS	Medical Expenditure Panel Survey
MEPS-HC	Medical Expenditure Panel Survey—Household Component
MEPS-IC	Medical Expenditure Panel Survey—Insurance Component
MEPS-MPC	Medical Expenditure Panel Survey—Medical Provider Component
US	United States
